# Cancer of unknown primary (CUP) through the lens of precision oncology: a single institution perspective

**DOI:** 10.1007/s00432-023-04741-y

**Published:** 2023-04-16

**Authors:** L. Weiss, K. Heinrich, D. Zhang, K. Dorman, K. Rühlmann, K. Hasselmann, F. Klauschen, J. Kumbrink, A. Jung, M. Rudelius, A. Mock, Steffen Ormanns, W. G. Kunz, D. Roessler, G. Beyer, S. Corradini, L. Heinzerling, M. Haas, M. von Bergwelt-Baildon, S. Boeck, V. Heinemann, C. B. Westphalen

**Affiliations:** 1 Department of Medicine III, University Hospital, LMU Munich, Munich, Germany; 2 Comprehensive Cancer Center Munich, University Hospital, LMU Munich, Munich, Germany; 3 German Cancer Consortium (DKTK), Partner Site Munich, Munich, Germany; 4 Institute of Pathology, Faculty of Medicine, LMU Munich, Munich, Germany; 5 Department of Radiology, University Hospital, LMU Munich, Munich, Germany; 6 Department of Medicine II, University Hospital, LMU Munich, Munich, Germany; 7 Department of Dermatology, University Hospital, LMU Munich, Munich, Germany; 8 Department of Dermatology, University Hospital Erlangen, Erlangen, Germany; 9 Department of Radiation Oncology, University Hospital, LMU Munich, Munich, Germany; 10 Bavarian Cancer Research Center (BZKF), Munich, Germany

**Keywords:** Precision oncology, Personalized medicine, Molecular tumor board, Cancer of unknown primary, CUP syndrome, Comprehensive genomic profiling

## Abstract

**Purpose:**

For patients with cancer of unknown primary (CUP), treatment options are limited. Precision oncology, the interplay of comprehensive genomic profiling (CGP) and targeted therapies, aims to offer additional treatment options to patients with advanced and hard-to-treat cancers. We aimed to highlight the use of a molecular tumor board (MTB) in the therapeutic management of CUP patients.

**Methods:**

In this single-center observational study, CUP patients, presented to the MTB of the Comprehensive Cancer Center Munich LMU, a tertiary care center, were analyzed retrospectively. Descriptive statistics were applied to describe relevant findings.

**Results:**

Between June 2016 and February 2022, 61 patients with unfavorable CUP were presented to the MTB, detected clinically relevant variants in 74% (45/61) of patients, of which 64% (29/45) led to therapeutic recommendation. In four out of 29 patients (14%), the treatment recommendations were implemented, unfortunately without resulting in clinical benefit. Reasons for not following the therapeutic recommendation were mainly caused by the physicians’ choice of another therapy (9/25, 36%), especially in the context of worsening of general condition, lost to follow-up (7/25, 28%) and death (6/25, 24%).

**Conclusion:**

CGP and subsequent presentation to a molecular tumor board led to a high rate of therapeutic recommendations in patients with CUP. Recommendations were only implemented at a low rate; however, late GCP diagnostic and, respectively, MTB referral were found more frequent for the patients with implemented treatment. This contrast underscores the need for early implementation of CGP into the management of CUP patients.

## Introduction

Cancer of unknown primary (CUP) comprises a heterogeneous group of metastatic malignancies, in which extensive clinical and diagnostic work-up including physical examination, radiological imaging, and histopathological investigation, does not lead to the identification of a primary site (Fizazi et al. 2015). While 3–5% of all malignancies are classified as CUPs, the underlying biology of these cancers remains mostly unknown (Pentheroudakis et al. 2007), and the prognosis for patients with CUP is generally poor with a median overall survival < 1 year (6–10 months) (Fizazi et al. 2015). The importance of a meticulous diagnostic work-up is underscored by the fact that it can identify a minority of CUP patients (15–20%), which can be attributed to a distinct clinical entity which derives benefit from site-specific treatment (Fizazi et al. 2015). The remaining 80–85% of patients belong to the group of unfavorable CUPs with a dismal prognosis.

Comprehensive genomic profiling (CGP) of tumor tissue is a powerful diagnostic tool to identify therapeutic targets in advanced cancers and might have the potential to determine the tissue of origin in patients with CUP (Oien and Dennis 2012). Several studies have demonstrated that molecular profiling may aid in identifying the tissue of origin (Bridgewater et al. 2008; Greco et al. 2013; Ye et al. 2020; Xu et al. 2016). However, there is conflicting evidence as to how this might translate into clinical benefit. In some reports, identification of the tissue origin by gene expression profiling has been reported to improve the survival of CUP patients by allowing more site-specific therapy to be administered rather than the empiric regimes that have been the standard approach (Greco et al. 2013; Hainsworth et al. 2013). In two randomized trials, identification of the tissue of origin did not lead to improved treatment outcome (Fizazi et al. 2019; Hayashi et al. 2019). Kato et al. implemented a molecular Matching Score (MS) that was defined as the number of alterations (not counting variants of unknown significance (VUS)) targeted by administered drugs divided by the total number of pathogenic alterations (not counting VUS) (Kato et al. 2022). Higher MS was the only factor that predicted significant improvement of survival for treated patients in post hoc univariate and multivariate analysis (Kato et al. 2022).

At CCCM^LMU^, patients with advanced cancers including CUP have access to CGP within a dedicated precision oncology program (Heinrich et al. 2023). To investigate the benefit with regard to the overall survival and rates of implementation of the MTB recommendation, we report clinical outcomes of a cohort of 61 patients with CUP syndrome.

## Materials and methods

This is a retrospective observational study, analyzing 61 patients with CUP diagnosis presented to the molecular tumor board at a tertiary care center (University Hospital Munich) between June 2016 and February 2022. CUP diagnosis was defined as a carcinoma or undifferentiated neoplasm for which a standardized diagnostic work-up failed to identify the primary tumor responsible for metastatic seeding (Krämer et al. 2023). Minimal standard diagnostic work-up for initial diagnosis included blood analysis, radiologic imaging, and detailed medical history. Patients were either diagnosed internally at internal departments of the University Hospital of LMU or referred by external partners mainly from practice-based settings. Charts, molecular profiles, and tumor board decisions were reviewed. Descriptive statistics were applied to describe relevant findings. The analysis was approved by the local ethics committee. The purpose of this study was to determine the feasibility and the impact of CGP and subsequent implementation of MTB recommendations in CUP patients.

The Munich Molecular Tumor board (MTB) consists of an interdisciplinary team of physicians and scientists with expertise in precision oncology. In this tumor board, oncologists, pathologists/molecular pathologists, tumor geneticists, and experts for precision oncology discuss CGP results within a patient’s clinical context. The MTB reviews results of molecular diagnostics, relevant tumor characteristics, and patient’s clinical course of disease, and aims to recommend a personalized treatment and/or further diagnostic procedures for each patient. Treating physicians decide on the timing for the presentation of a case to the MTB and can also register patients with external CGP. Treatment recommendations are supported by the levels of evidence for molecular targets in accordance with the European Society for Medical Oncology (ESMO) Scale for Clinical Actionability of Molecular Targets and according to a harmonized German scale (Leichsenring et al. 2019). To support the evaluation and interpretation of CGP results, an on-site literature database was created. The clinical implementation of the recommendations remains the responsibility of the treating physician.

### Workflow

Extended molecular testing was initiated by the organ-/entity-specific interdisciplinary tumor board or after consultation with the team of the precision oncology program. Several patient characteristics can help identify patients that might benefit from CGP (Heinrich et al. 2023):

- Patients suffering from advanced disease with no further “standard of care” therapeutic options.
- Patients with an unusual clinical presentation or disease course for the respective disease or suffering from a rare pathological subtype.
- Patients with a performance status and life expectancy allowing to potentially benefit from the MTB recommendation.

Cases were submitted to the MTB via an online registration system based on the Clinical Workplace Program of the hospital. Clinical data are entered by treating physician.

### Diagnostics and patients

**Fig. 1:**
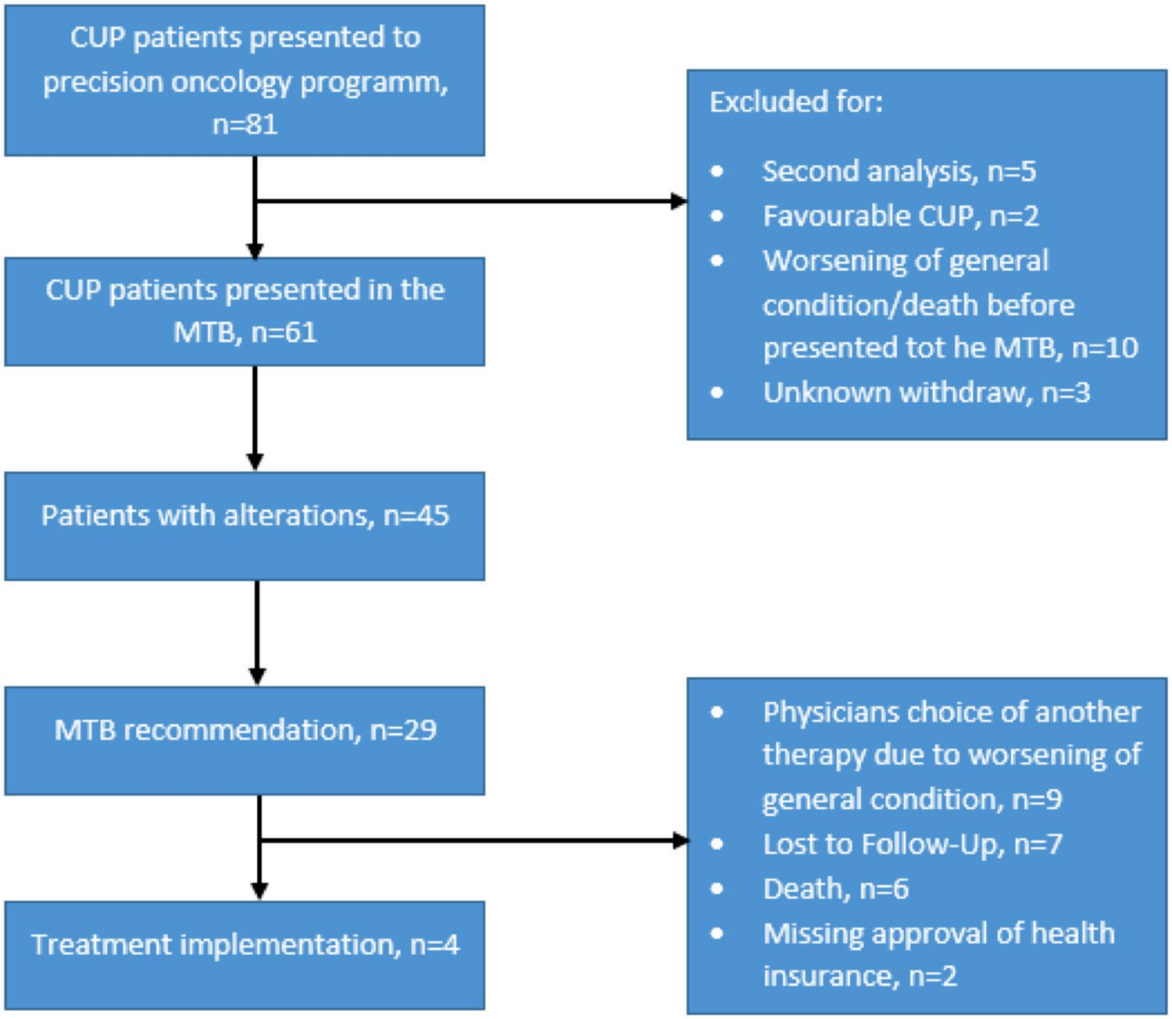
Consort diagram of the CUP patients within the MTB. Second analysis was defined as an unsuccessful first attempt of CGP, with a successful second attempt. Favorable CUP was defined by obvious analogies to certain cancers with a known primary (Krämer et al. 2023)

At the CCC^LMU^, different types of extended molecular diagnostic tests have been used, most of them available through the local department of pathology. The following tests were used in routine clinical practice: Oncomine focus (50 genes), Oncomine comprehensive v3 (161 genes), TSO500 (Illumina, DNA level: 525 genes, RNA level: 53 genes, signatures: TMB), or a combination of OCAplus (Ion Torrent: DNA level: 501 genes, signatures: TMB) and Archer Oncology Research (RNA level: 74 genes). In some cases, testing was performed by commercial providers. In-house testing can be performed on tumor tissue (FFPE) or on liquid biopsies. Patients in which CGP had already been performed can also be referred to the MTB from external hospitals or physicians. Next-generation sequencing (NGS) data were analyzed and medical records were reviewed retrospectively to evaluate clinical characteristics. Between June 2016 and February 2022, 81 patients with CUP were presented to the MTB team for case discussion. 20 patients were excluded from the analysis due to double registration (initial NGS-analysis was technically unsuccessful), favorable CUP (clinical–pathological subset with a more favorable prognosis), worsening of general condition or death before presentation in the MTB and other non-specified reasons (Fig. 1).

### Data analysis

To determine the clinical impact of molecularly guided therapies, we analyzed the follow-up for these patients. Cut-off date for follow-up analysis was February 1, 2022. If exact dates were missing, we set these on the 1st of the known month. If data regarding survival status are missing, we used last day of follow-up. All statistical analyses were performed using SPSS version 25 for Windows (SPSS Inc., Chicago, IL). The survival curves were analyzed by the Kaplan–Meier method and described by median values. Comparisons of survival-based outcomes were conducted using log-rank tests and Cox regression analyses that were described as hazard ratios with 95% confidence intervals (95% CI). *p* values < 0.05 were considered statistically significant. To compare the means of two independent groups, we used the independent samples *t* test.

## Results

Until February 2022, a total of 81 patients with CUP were presented to the precision oncology program. 20 CUP patients were excluded from the analysis. Five patients underwent CGP, but the initial molecular analysis was unsuccessful. After successful re-testing, patients were discussed in the MTB. We excluded two patients with favorable CUP. Ten patients presented with very poor performance status and did not undergo CGP. Three patients were withdrawn by the clinical team for unknown reasons. After exclusion of these patients, a total of 61 patients with CUP were discussed in the MTB and were included in this single-center experience.

### Patient characteristics

Patient characteristics are presented in Table 1. The median age of patients was 60 years (range: 23–85 years). 28 patients were referred from external partners, and in 33 patients, the diagnosis was made at LMU hospital. There was a slight imbalance of genders (57.4% male patients (35/61), 42.6% female patients (26/61)). Regarding histopathology results, most patients (*n* = 51 (83.6%)) were diagnosed with adenocarcinoma, four (6.6%) with squamous cell carcinoma, two (3.3%) patients with neuroendocrine carcinoma, one (1.6%) patient with melanoma, and four (6.6%) patients with other histology including urothelial carcinoma and small-cell cancer or missing information due to external analysis, respectively. Molecular diagnostics were performed for every patient.

**Table 1:** Patient baseline characteristics

Demographic variable	MTB
	*N* = 61
Age, years	
Median	60
Range	23–85
Gender, *n* (%)	
Men	35 (57.4)
Women	26 (42.6)
Panel, *n* (%)	
Oncomine^a^	47 (77.0)
FMI^b^	9 (14.8)
Other	5 (8.2)
Tissue origin, *n* (%)	
Adenocarcinoma	51 (83.6)
Sarcomatoid carcinoma	5 (8.2)
Adenosquamous carcinoma	4 (6.6)
Squamous cell carcinoma	4 (6.6)
Neuroendocrine carcinoma	2 (3.3)
Others (urothelial cancer, small-cell cancer, and melanoma)	4 (6.6)
Alterations, *n* (%)	
Yes	45 (73.8)
No	16 (26.2)
MTB recommendation, *n* (%)	
Yes	29 (64.4)
No	16 (35.6)
Implementation of recommended therapy, *n* (%)	
Yes	4 (13.8)
No	25 (86.2)
Number of previous therapy lines, *n* (%)	
0	14 (23.0)
1	25 (41.0)
2	7 (11.5)
3	3 (4.9)
4	1 (1.6)
Unknown	11 (18.0)

^a^Oncomine Focus Panel, 50–525 genes (DNA)

^b^Foundation One CDx (FMI) is a single tissue-based test designed by Roche, analyzing 324 genes (DNA)

Patients had received a median of one (range 0–4 therapies) previous therapies prior to MTB presentation. Median turnaround time from initial diagnosis to MTB referral was 249 days (28/61, 45.9%) for external patients 79 days (33/61, 54.1%) for patients with determination of initial diagnosis at our hospital, respectively.

### MTB discussion and treatment recommendations

All 61 patients were discussed in the MTB. At least one molecular alteration was found in 45 patients (45/61, 74%). In the remaining 16 patients, no genomic alterations were detected (16/61, 26%).

29 out of 45 patients had clinically relevant, actionable alterations and received a therapeutic recommendation by the MTB with the following ESCAT-Scale range IC (1/29, 3.4%), IIIB (7/29, 24.1%), IIIA (17/29, 58.6%) and others including preclinical data (3/29, 10.3%) (Table 2).

**Table 2:** Therapeutically relevant alterations in the cohort

Pat-Nr	Target	Therapy	NCT	ESCAT
8	NF2 mutation	mTOR inhibitor	2B	IIIB
9	STK11 mutation	mTOR inhibitor	2C	IIIB
14	TMB high	Immunotherapy	1C	IIIA
17	TMB high	Immunotherapy	1C	IIIA
18	TMB high	Immunotherapy	1C	IIIA
19	KRAS mutation	Trametinib/hydroxychloroquine	1C	IIIB
23	ErbB2 mutation	Trastuzumab/lapatinib	1A	IIIA
25	PIK3CA mutation	mTOR inhibitor	2C	IIIB
26	IDH2 mutation	Enasidenib	2A	IIIA
28	HER2 & HER3 mt	HER-directed therapy	2C	IIIB
33	EML-ALK	Alk-inhibitor	1C	IIIA
43	TMB high	Immunotherapy	1C	IIIA
44	AR mutation	Antiandrogen	2C	IIIA
47	NRAS mutation/TMB high	Immunotherapy	1C	IIIA
50	ErbB2 mutation	pan-Her TKI	2C	IIIA
51	BRAF V600E	BRAF/MEK inhibitor	1C	IC
54	FGFR1 mutation	FGFR1 directed therapy	2C	IIIB
55	TMB intermediate + PD-L1 pos	Already received immunotherapy	N/A	N/A
61	CDK4 amplification	CDK4/6 inhibitor	2B	IIIA
62	PIK3CA mutation	mTOR inhibitor	2C	IIIB
65	PIK3CA, IDH1	mTOR inhibitor, Ivosidenib	2A	IIIA
66	FGFR3 mutation	FGFR inhibitor	3	IV
67	FGFR2-fusion	Pemigatinib	2A	IIIA
68	MSI	Immunotherapy	1C	IIIA
70	BRAF mutation	Sorafenib/trametinib	2C	IIIA
72	TMB high	Immunotherapy	1C	IIIA
74	PIK3CA	mTOR inhibitor	2A	IIIA
76	mTOR/AKT	Everolimus/exemestane	2B	IIIA
79	ARID1A	Immunotherapy	2B	IIIA

Most commonly, the MTB recommended the use of immunotherapy with checkpoint inhibitors (8/29, 28%), followed by mTOR-inhibitors with 6 out of 29 recommendations (21%). The most common genomic alterations leading to a recommendation were high tumor mutational burden TMB^high^ (17.2%) and activating alterations in PIK3CA (13.8%) and ERBB2 (10.3%). The molecular profiles observed did not lead to the identification of primary side in our cohort.

### Survival analysis

**Fig. 2:**
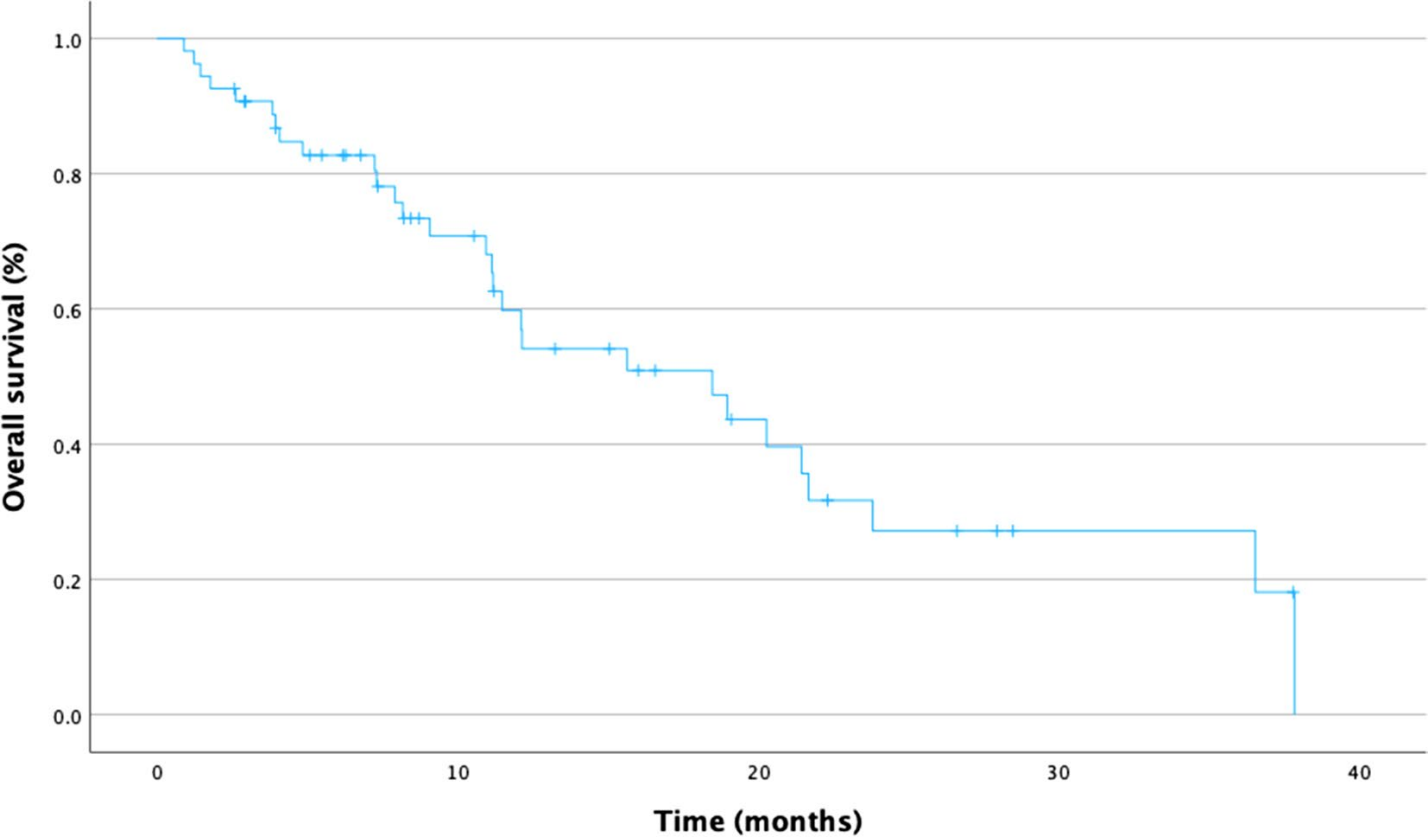
Analysis of median Overall survival, *n* = 61

Follow-up information was available for 54 out of 61 patients (88.5%). Median overall survival in the whole cohort was 18.5 months (range 0.9–51.7 months) (Fig. 2). The survival after discussion in the MTB was 3.7 months (range 0–34.0 months).

### Implementation of MTB recommendation

Four out of 29 patients were treated accordingly to the MTB recommendation off-label (mTOR inhibitor, IDH2-inhibitor, ALK inhibitor, BRAF/MEK inhibitor). After the start of recommended treatment, three out of four patients discontinued treatment due to worsening of general condition, one patient died with progressive disease while being treated with the recommended treatment. All patients died on average within 3 months and none of the patients reached the first follow-up restaging. For further information, please refer to Table 3.

**Table 3:** Clinical course of patients treated according to MTB recommendation

Pat-Nr	Target	Other alterations	Recommendation	Therapy	Start of recommended treatment	Discontinuation	Death	Reason of discontinuation
9	STK11 Mutation	KDR Amplification, KIT Amplification, BCORL1 Alteration, CDKN1B Deletion, SMARCA4 Alteration, TP53 Alteration	mTOR	Everolimus	6/1/2020	9/1/2020	12/15/2020	Worsening of general condition
26	IDH2 Mutation	None	IDH2-Inhibitor	Enasidenib	2/1/2020	4/11/2020	4/11/2020	Death
33	EML-ALK	None	ALK-Inhibitor	Ceritinib	11/29/2017	1/15/2018	1/25/2018	Worsening of general condition
51	BRAF V600E	None	BRAF/MEK-Inhibitor	Dabrafenib/Trametinib	1/22/2018	3/23/2018	3/30/2018	Worsening of general condition

Reasons for not implementing therapeutic recommendations were physicians’ choice favoring an alternative therapy (9/25, 36.0%) in the context of worsening of general condition, patients being lost to follow-up (7/25, 28.0%), death (6/25, 24.0%), missing approval of health insurance (2/25, 8.0%), and other medical reasons (1/25, 4.0%).

We compared the group of patients who received a treatment recommendation without implementation of the MTB (*n* = 25), and patients who were treated with recommended treatment (*n* = 4) regarding several time points of clinical history. We did not find statistically significant difference in the median time from initial diagnosis to the first treatment (including surgery, radiation, and systemic therapy), time from initial diagnosis to CGP registration, length of CGP diagnostics, time from initial diagnosis to MTB recommendation, and time from MTB recommendation to death. When median days from CGP results to MTB presentation were compared, the “other treatment” group was in favor with 12 days, compared to 36 days in the group of patients with implemented treatment (*p* = 0.011) (Table 4).

**Table 4:** Relevant time spans in patients receiving any treatment after MTB discussion

	MTB recommended treatment, *n* = 4	Other treatment, *n* = 25	*t* test, *p* value
Days from initial diagnosis to first treatment	31 (12–86)	32 (0–245)	0.613
Days from initial diagnosis to CGP registration	262 (19–434)	167 (0–925)	0.851
Turnaround time for CGP	13 (8–28)	15 (9–32)	0.697
Days from Test results to MTB referral	36 (33–47)	12 (4–80)	0.011
Days from MTB to implementation of recommended therapy	69 (14–124)		
Days to MTB referral	312 (82–486)	200 (46–964)	0.951
Days from MTB to death	137 (80–281)	117 (11–523)	0.791
Average number of previous therapies at MTB referral	2 (0–3)	1 (0–3)	0.141

## Discussion/conclusion

Fundamental advances have been made in the diagnostic and therapeutic management of cancer patients due to the implementation of NGS and targeted therapies. The management of CUP patients with poor prognosis remains challenging despite the availability of a variety of cytotoxic chemotherapy regimens (Goodman et al. 2018). The implementation of CGP and CUP patients promises changes in treatment outcome and has therefore been included into international guidelines (Krämer et al. 2023).

In our cohort, a total of 81 CUP patients were referred to the precision oncology program and the 61 patients that were presented to the MTB are discussed in this manuscript. Of note, patients with favorable CUP were excluded from this analysis as they should be treated in accordance with the assumed primary site. In most cases, prognosis of this subgroup compares to the assumed primary cancer (Fizazi et al. 2015). Ten patients were not discussed in the MTB due to worsening of general condition or death.

Cancer of unknown primary is defined as a carcinoma or undifferentiated neoplasm for which a standardized diagnostic work-up failed to identify the primary tumor responsible for metastatic seeding (Krämer et al. 2023) Although sarcomas, melanomas, germ cell tumors, hematological malignancies, and neuroendocrine tumors with unknown location of the primary tumor are by current definition not included in the CUP definition, we included one patient with melanoma and two patient with neuroendocrine carcinoma (Prasad et al. 2010; Perren et al. 2017).

Median age of patients with CUP was 60 years representing the known age range of initial diagnosis (Losa et al. 2018; Pavlidis and Pentheroudakis 2012).

In this cohort, 74% of patients with CUP receiving CGP harbored a genomic alteration. Of those, 64% carried a clinically relevant alteration. Current data show the complex molecular profile of CUP patients with a median of one alteration per tumor (Ross et al. 2015). A low rate of implementation (17%) of previous MTB recommendations (41.4%) was reported for the first 1000 patients of our precision oncology program between 2016 and 03/2020 (Heinrich et al. 2023). Early detection of targetable alterations via CGP could open a new range of innovative therapies with the goal to increase overall survival (Massard et al. 2017). Although specific gene expression profiles are currently recognized in cancers from different sites of origin, reflecting typical specific genomic patterns of different tumor entities, we were not able to identify the primary site of origin in our cohort based on the gene expression profile (Hainsworth et al. 2012; Kato et al. 2021).

The 61 patients presented in the MTB were previously treated with a median of one line of systemic therapy. Considering the poor survival prognosis of less than one year and the minimal benefit of cytotoxic chemotherapy, new strategies regarding targeted therapy should be considered as early as possible (Fizazi et al. 2015; Hannouf et al. 2018). Therefore, the European Society of Medical Oncology (ESMO) recommends the use of NGS in the setting of dedicated precision oncology programs for hard-to-treat cancers (Mosele et al. 2020). In our cohort, 23% of our patients received CGP at initial diagnosis.

In our experience, there were considerable differences in between time to referral to the molecular tumor board in between patients diagnosed at LMU hospital when compared to external patients. Given the fact, that reduced performance status and elevated lactate dehydrogenase (LDH) are negative prognostic factors for overall survival in CUP patients (good prognostic group: ECOG 0 or 1 and normal LDH, poor prognostic group: ECOG > 1 or elevated LDH), time to treatment should be as short as possible (Fizazi et al. 2015; Qaseem et al. 2019; Tomuleasa et al. 2017).

Unprecedented advances have been made in cancer treatment with the use of immune checkpoint inhibitors (ICI) (Morad et al. 2021). This class of cancer therapeutics has led to a fundamental shift in the management of cancer and became therapeutic standard in various tumor entities. Several clinical features or respective biomarkers seem to predict the response to ICI, this includes patients with dMMR/MSI^high^ cancers (Marabelle et al. 2020), tumors with high tumor mutational burden (TMB) ≥ 10 mutations/Mb (Brahmer et al. 2012), or PD-L1 overexpression or amplification (Ott et al. 2018). In our cohort, five out of 45 patients were classified as TMB high (≥ 17 mutations/mb), six patients as TMB intermediate (3–16 mutations/mb), and four additional patients displayed PD-L1 positivity while one patient was found to be dMMR/MSI^high^.

Furthermore, seven patients were found to carry alterations in ERBB2 (HER2) and PIK3CA.

In the setting of biomarker driven diagnostic, HER2 has been recognized as an important therapeutic target in various malignancies including breast, colorectal, gastric, and biliary tract cancers (Iqbal and Iqbal 2014). On this basis, a variety of novel HER2-targeted drugs are under development, and related clinical trials are ongoing (Cutsem et al. 2015; Zhu et al. 2021; Modi et al. 2022; Ross et al. 2018; Koeberle and Fritsch 2021). Thus, the finding that a fraction of CUP patients present with HER2-positive disease might carry clinical significance. Targeting PIK3CA mutant cancers is complex (Samuels and Waldman 2010). While SOLAR-1 showed significant benefit of PIK3CA mutant, hormone receptor positive breast cancer treated with Alpelisib/Fulvestrant, broad activity of PIK3CA inhibitors has not been seen across cancers. Nevertheless, within the MOSCATO-1 trial, responses to PIK3CA-directed therapies in the setting of advanced cancers have been observed (Massard et al. 2017; Baselga et al. 2018; Verlingue et al. 2017). Accordingly, in patients with limited therapeutic options and in the absence of other strong oncogenic drivers or molecular targets, PIK3CA might constitute a therapeutic option.

In our cohort, median survival was 18.5 months, which is slightly above published survival in unfavorable subtype CUP patients (Fizazi et al. 2015; Culine et al. 2002). Follow-up information was available for 54 out of 61 patients. In only four out of twenty-nine patients’ treatment, recommendations were implemented; however, none of the patients derived clinical benefit from the intervention. All four patients died within three months after implementing targeted therapy. Published data suggest that CUP patients receiving targeted therapy can derive clinical benefit (Hainsworth et al. 2013; Kato et al. 2021, 2022). Due to the small number of patients with implemented treatment recommendation, we are not able to prove or refute this statement (Table 4). It should be noted however that most patients in our cohort presented at (very) advanced stages and a significant proportion of patients were medically unfit to receive any, let alone experimental treatment. It seems conceivable that this fact led to the disappointing results presented here. In this regard, it is noteworthy that the four patients receiving experimental treatment had a comparatively short survival (mOS 9.1 vs 18.6 months, *p* = 0.421) when compared to patients who received alternative treatment arguing for an especially aggressive clinical course.

Aside from the challenges and discouraging results presented here, we still believe that CGP should be standard of care in the management of CUP patients. Rather than using it in the further or last-line setting, CUP patients should have access as early as possible to identify those with potential benefit of targeted treatments and to allow screening for clinical trials (Pauli et al. 2021). Otherwise, as demonstrated in this study, a potentially effective treatment cannot be implemented due to rapid worsening of general condition. Overall, more standardized, guideline-adherent management of patients with CUP is imperative to achieve better outcomes in this group of patients with high unmet medical need.

## Data Availability

All authors had access to the data published in this paper.
